# Large and Small Dendritic Spines Serve Different Interacting Functions in Hippocampal Synaptic Plasticity and Homeostasis

**DOI:** 10.1155/2016/6170509

**Published:** 2015-12-31

**Authors:** Joshua J. W. Paulin, Peter Haslehurst, Alexander D. Fellows, Wenfei Liu, Joshua D. Jackson, Zelah Joel, Damian M. Cummings, Frances A. Edwards

**Affiliations:** Department of Neuroscience, Physiology and Pharmacology, University College London, London WC1E 6BT, UK

## Abstract

The laying down of memory requires strong stimulation resulting in specific changes in synaptic strength and corresponding changes in size of dendritic spines. Strong stimuli can also be pathological, causing a homeostatic response, depressing and shrinking the synapse to prevent damage from too much Ca^2+^ influx. But do all types of dendritic spines serve both of these apparently opposite functions? Using confocal microscopy in organotypic slices from mice expressing green fluorescent protein in hippocampal neurones, the size of individual spines along sections of dendrite has been tracked in response to application of tetraethylammonium. This strong stimulus would be expected to cause both a protective homeostatic response and long-term potentiation. We report separation of these functions, with spines of different sizes reacting differently to the same strong stimulus. The immediate shrinkage of large spines suggests a homeostatic protective response during the period of potential danger. In CA1, long-lasting growth of small spines subsequently occurs consolidating long-term potentiation but only after the large spines return to their original size. In contrast, small spines do not change in dentate gyrus where potentiation does not occur. The separation in time of these changes allows clear functional differentiation of spines of different sizes.

## 1. Introduction

Dendritic spines form the postsynaptic element of most excitatory synapses in the mammalian cortex and hippocampus and their differing sizes and morphologies are directly related to synaptic strength [1]. The strength of spine synapses is highly plastic which is important for homeostatic protection from excitotoxicity but also for the laying down and retrieval of memory [2–4]. Being directly related to the strength of synapses, it is not surprising that the size of spines also changes with plasticity of synaptic transmission [5–7]. However, it remains controversial whether the diversity of spine morphologies represents a continuum, with size simply reflecting the history of the synapse or rather that spines with different morphological classifications represent different functional entities. To address this question, we investigate how different spines react and interact when they are strongly and simultaneously stimulated across the network. Application of tetraethylammonium chloride (TEA) results in “chemical long-term potentiation” (LTP) at CA3-CA1 synapses [8] and has been shown to cause growth in a subset of small spines when imaged 2 hours after induction [9]. However, such global stimulation would also be expected to cause an immediate protective homeostatic response due to both massive depolarisation and resulting glutamate release. Moreover, possible effects of the osmolarity change caused by adding 25 mM TEA must be considered, a control that has seldom been included in previous studies. Here, we report that, in response to TEA, not only the direction but also the time course of changes in the response of larger and smaller spines can be separated. Moreover, in DG granule cells, where TEA does not cause long-term potentiation [10], the response of spines differs from that of CA1 pyramidal cells confirming the functional link between spine size and synaptic plasticity.

## 2. Material and Methods

### 2.1. Animals and Slices

Organotypic slices were prepared using standard methods [11] from 5- to 6-day-old mice of either sex expressing green fluorescent protein (GFP) on the* Thy1* promoter (GFPS mice) [12], resulting in a subset of their glutamatergic neurones being fluorescent. Organotypic slices used for granule cell imaging and electrophysiological recording were made with the standard protocol of parasagittal sections. For imaging of CA1 pyramidal cells, slices were angled as for preparation of acute slices for electrophysiological recording (~15° off parasagittal) as this maintains more CA1 neurones intact and ensures that the preparations for imaging and recording were as similar as possible.

Acute slices were made using standard methods [13] adapted for mouse [14]. Each hemisphere was sectioned (400 *μ*m) in ice-cold dissection artificial cerebrospinal fluid (ACSF) containing (in mM) 125 NaCl, 2.4 KCl, 26 NaHCO_3_, 1.4 NaH_2_PO_4_, 20 D-glucose, 3 MgCl_2_, and 0.5 CaCl_2_, pH 7.4, ~315 mOsm/L. The hippocampus with a portion of entorhinal cortex was dissected and placed into a chamber containing bubbled dissection ACSF at room temperature (~20°C). After 5 minutes, the chamber was warmed to 35°C. Slices were then, at 5-minute intervals, consecutively transferred to increasingly physiological Ca^2+^ and Mg^2+^ ion concentration (in mM): (i) 1 Mg^2+^, 0.5 Ca^2+^; (ii) 1 Mg^2+^, 1 Ca^2+^; (iii) 1 Mg^2+^, 2 Ca^2+^ (standard ACSF). After 20 minutes at 35°C, slices were allowed to return to room temperature for at least 40 min before recording.

All animal procedures were performed in compliance with the United Kingdom Animals (Scientific Procedures) Act 1986.

### 2.2. Chemical LTP

TEA (25 mM) dissolved in ACSF was bath perfused (~1 mL/min) for 5 minutes before returning to standard ACSF [8]. For time control experiments TEA was not included but experiments were otherwise identical with and without TEA. In osmolarity control experiments, the protocol was identical but 50 mM sorbitol replaced the TEA.

### 2.3. Imaging and Analysis

**Figure 1:**
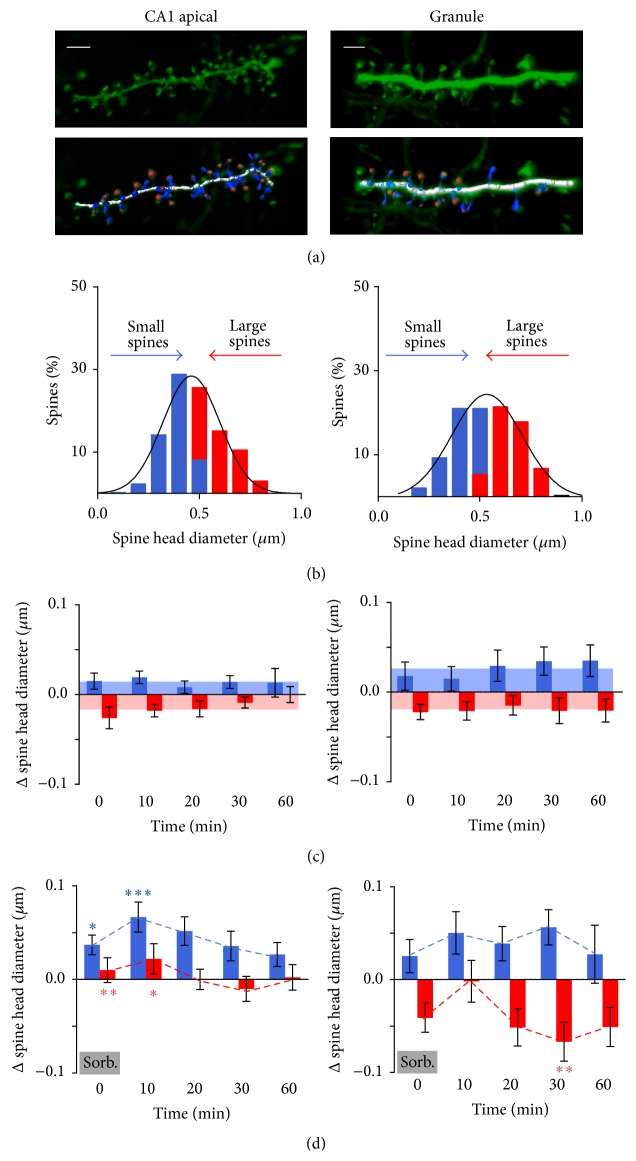
Control data from CA1 pyramidal cells or DG granule cells tend to fluctuate towards the mean but are similar over time (left panels: CA1 pyramidal cells; right panels: DG granule cells). (a) 3D reconstruction of deconvolved confocal images of sections of dendrite of a hippocampal organotypic slice and model of the dendritic spines as superimposed by Imaris. Scale bar: 2 *μ*m. Note one large spine not modelled by Imaris (see Section 2). (b) Before application of TEA, estimated spine head diameters are normally distributed. Small (blue) and large (red) spines are defined as spines with diameters less or greater than the mean diameter, respectively. (c) Change in spine diameter after repeated imaging in control ACSF. Limits of the shaded region represent the mean of all time points for small (blue) and large (red) spines. (d) Change in spine diameter after repeated imaging following transient high osmolarity ACSF perfusion (50 mM sorbitol, 5 min). Dotted line represents mean change of small (blue) and large (red) spines at that time point. Note the data at time 0 are sampled during the sorbitol wash-in.* Post hoc* analysis of control data versus sorbitol (3-way interaction between size, time, and treatment): ^*∗*^ *p* < 0.05; ^*∗∗*^ *p* < 0.01; ^*∗∗∗*^ *p* < 0.001.

Dendrites chosen at random were scanned (confocal microscope: Olympus Fluoview 300 or Zeiss LSM 510; Olympus 60x water immersion objective, N.A. 0.9) at 6x gain with 0.2 *μ*m steps. The microscope used did not affect the results. For maximum resolution, all imaging experiments were carried out in organotypic hippocampal slices (2-3 weeks* in vitro*) at 30 ± 1°C. After deconvolution (AutoQuant, Media Cybernetics), images were reconstructed in 3D using the Filament Tracer module of Imaris (Bitplane) to estimate spine diameter. Filament Tracer estimates the diameter of a sphere equivalent to the volume estimated from several automatically defined sections of the spine taken through the* z*-plane (Figure 1(a)). Thus, the “diameters” reported are not a direct measure (which would be beyond the resolution of the image) but rather a back extrapolation from several images estimating the overall 3D head volume. This calculated value, rather than being an accurate absolute measure of the diameter of the active zone, is a high-resolution method of comparing changes in individual spines across time, while avoiding the assumption of where on the spine the synaptic contact would be situated. Moreover, using diameter rather than volume transforms the skewed volume data to a normal distribution, facilitating analysis. All image analysis was carried out blind to treatment and to the time point of the experiment. Data in figures are presented for spines in which size could be reliably estimated at the initial control time point (−10 minutes, to which all other time points were compared) and at least 3 of the 5 postinduction time points (diameter >* z*-interval, ensuring at least two intersecting planes). Note that this excludes the smallest spines. In some cases, we were also unable to model the largest spines, so they were also excluded. This may relate to irregularities in the shapes of spines being better resolved in these cases and so causing problems with the algorithm used by Imaris (e.g., see Figure 1(a), granule cell dendrite). The time course and direction of change for both small and large spines in both CA1 pyramidal cells and dentate gyrus (DG) granule cells were consistent whether all spines were considered individually or the results were averaged by experiment. Results shown are for individual spines independent of preparation.

### 2.4. Electrophysiology

Field recording is the method of choice for measuring effects over the network and for avoiding effects of cell dialysis in LTP experiments. However, as the CA1 cell layer tends to spread out in organotypic slices, the interface between the cell body layer and dendrites becomes too diffuse for recording field excitatory postsynaptic potentials (fEPSPs). We have previously demonstrated however that the morphology of dendritic spines in CA1 is very similar in acute and organotypic preparations [15, 16] and so fEPSPs were recorded in acute slices from 4-week-old male mice, being the most similar preparation suited to these recordings. In the case of dentate gyrus, the cell layer is often less affected by the spread of the cell layers over time and so in some preparations it is possible to obtain field recordings. We have thus concentrated on acute slices throughout the field experiments but compared the dentate results to organotypic slices where possible.

Slices were transferred as needed to a heated (30 ± 1°C), submerged chamber, perfused with ACSF, and allowed to recover for 1 h in the recording chamber. A glass stimulating electrode (filled with ACSF, resistance 1–3 MΩ) was positioned in the appropriate projection (stratum radiatum or alternately medial or lateral perforant path). A glass recording electrode (filled with ACSF, resistance 1–3 MΩ) was positioned in stratum radiatum of CA1 or in the molecular layer of the dentate gyrus to record a dendritic field potential. Stimulation intensity was set at ~50% of the intensity required to evoke a population spike and recording continued until a 15-minute stable baseline was achieved. LTP conditioning consisted of either application of TEA (as above) or 3 trains of tetani, each consisting of 20 pulses at 100 Hz, 1.5 s intertrain interval, and recording (0.1 Hz) was then continued for another 60 minutes. Data are displayed as averages of 6 consecutive responses over 1 minute. Recording and analysis were carried out using WinWCP synaptic analysis software (Dr. John Dempster; http://spider.science.strath.ac.uk/sipbs/software.htm).

### 2.5. Statistics

Statistics were performed using SPSS (Version 23) or Graphpad Prism (Version 6). All data are expressed as means ± SEM. For analysis of spine head size, a Generalised Linear Mixed Model was used to compare control versus sorbitol, control versus TEA, and sorbitol versus TEA in each of the two cell types. Repeated measures of the change in size over time (using unstructured covariance) on each spine compared to the pretreatment time point took into account the different preparations for each treatment group. A 3-way comparison was made using time, size, and treatment as fixed effects. Reported probabilities refer to the* post hoc* analysis of the 3-way interactions using sequential Sidak adjustment for multiple comparisons. A robust estimate was used for missing data points. (As outlined above, data were included if reliable estimates could be obtained before treatment and for at least 3 of the subsequent 5 time points.)

All other analyses used paired or unpaired *t*-tests as appropriate.

## 3. Results

Using confocal microscopy, stretches of hippocampal dendrites were repeatedly scanned, reconstructed in 3D, and modelled (Figure 1(a)) at 10-minute intervals before (−10 min), during (0 min), and at several time points after (10, 20, 30, and 60 min) exposing the slice to TEA or sorbitol (osmotic control) or at the same time points with no change of solution (time control).

### 3.1. Controls

Spines were classified in terms of size and location. Estimated spine head diameters on apical dendrites of CA1 pyramidal cells (0.49 ± 0.006 *μ*m, *n* = 396) were significantly lower than for DG granule cells (0.53 ± 0.009 *μ*m, *n* = 280; Student's *t*-test *p* < 0.0001 versus CA1 apical spines). Spines were thus divided into those smaller or larger than 0.49 *μ*m for CA1 apical and 0.53 *μ*m for DG granule cells and this formed the initial distribution (designated −10 min, Figure 1(b)). Each spine was then compared to its own initial value over time (0, 10, 20, 30, and 60 min; Δ spine head size). (Note that using 0.53 *μ*m versus 0.49 *μ*m as the size threshold for DG granule cells made no qualitative difference to the result.)

**Figure 2:**
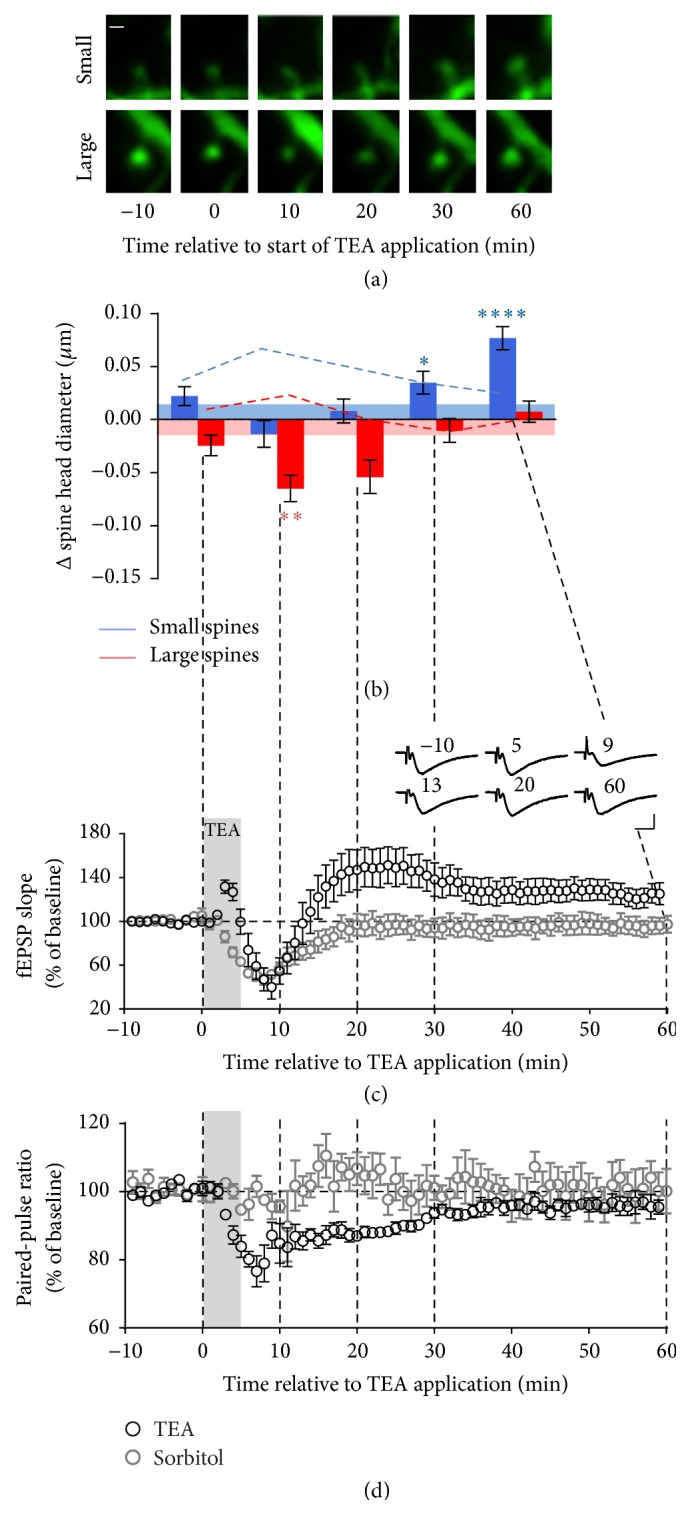
Large and small dendritic spines in CA1 pyramidal cells respond to TEA with different time courses corresponding to different phases of the synaptic response. (a) Typical example of a small and a large spine imaged before (−10), during (0), and after washout (10–60 min) of TEA (25 mM, 5 min). Scale bar: 0.5 *μ*m. (b) Quantification of changes in spine size relative to the pre-TEA measurement. Small spines, blue; large spines, red. Limits of the shading represent the mean change across time in the absence of TEA (time course control experiments). The dotted lines represent the mean change at each time point in response to sorbitol (osmolarity control experiments).* Post hoc* analysis of control data versus TEA (3-way interaction between size, time, and treatment): ^*∗*^ *p* < 0.05; ^*∗∗*^ *p* < 0.01; ^*∗∗∗∗*^ *p* < 0.0001. (c) fEPSP_slope_ and (d) PPR recorded in the CA1 region of acute hippocampal slices in response to stimulation of the Schaffer collateral before, during, and after application of TEA (black symbols) or sorbitol (grey symbols) as above. Error bars: SEM. Grey shading: TEA perfusion. Inset: averages of fEPSPs recorded from a typical slice over 1 min at 10 s intervals at the time indicated (min). Scale bar: 1 mV, 10 ms.

**Figure 3:**
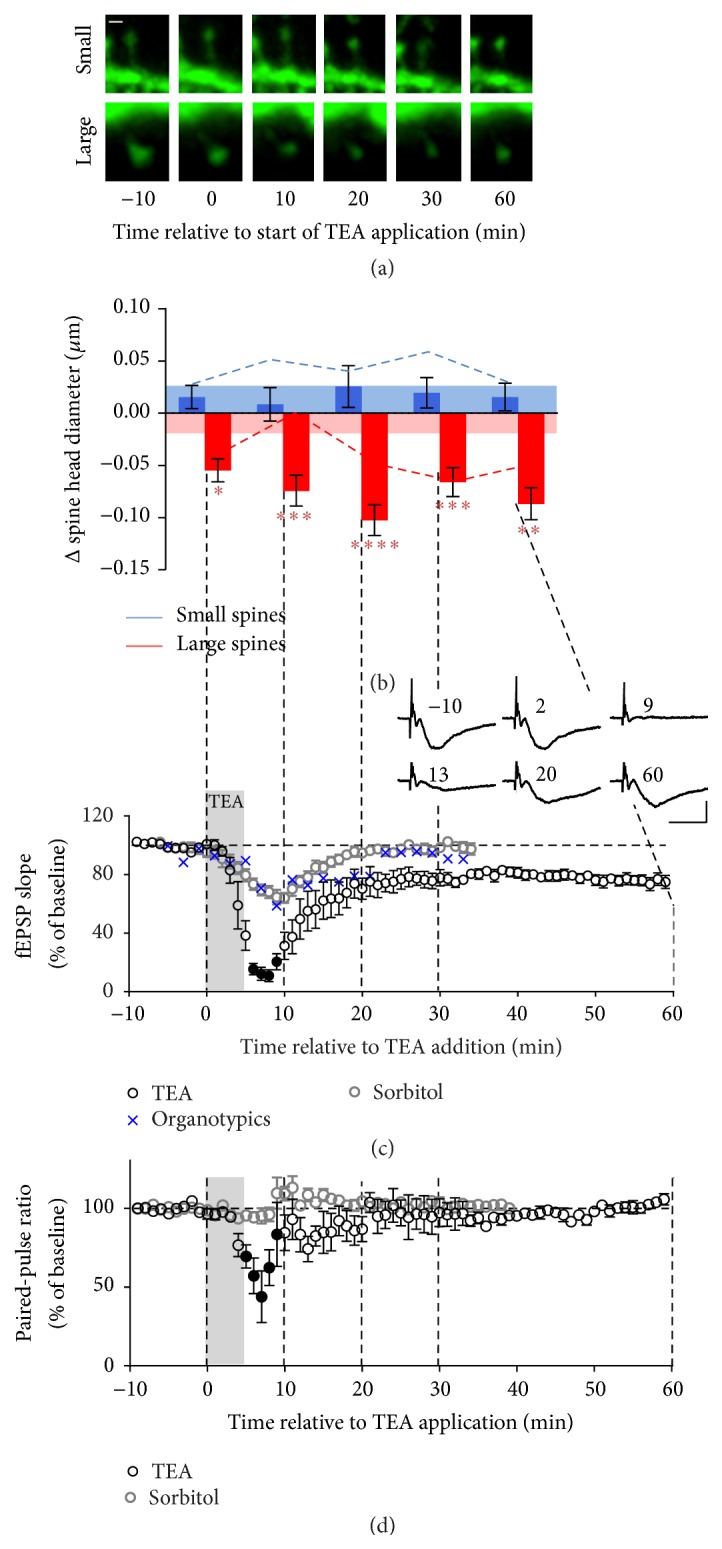
Synapses in DG granule cells behave differently from CA1 synapses in response to the same TEA stimulus. (a) Typical example of a small and a large spine imaged before (−10), during (0), and after washout (10–60 min) of TEA (25 mM, 5 min). Scale bar: 0.5 *μ*m. (b) Quantification of changes in spine size. Small spines, blue; large spines, red. Limits of the shading represent the mean change across time in the absence of TEA (time course control experiments).* Post hoc* analysis of control data versus TEA (3-way interaction between size, time, and treatment): ^*∗*^ *p* < 0.05; ^*∗∗*^ *p* < 0.01; ^*∗∗∗*^ *p* < 0.001; ^*∗∗∗∗*^ *p* < 0.0001. ((c), (d)) Slope and PPR of fEPSP recorded in the DG granule cells region in organotypic and acute hippocampal slices in response to stimulation of the perforant path, before and after application of TEA (black), as above, and high osmolarity ACSF (grey), as above. Closed symbols represent points where responses were too small for reliable measurement. Grey shading: TEA perfusion. Error bars: SEM. Inset: averages of fEPSPs recorded from a typical slice over 1 min at 10 s intervals at the time indicated (min). Scale bar: 1 mV, 10 ms.

*Effects of Time and Natural Fluctuation*. As would be expected from random fluctuation [17], in control experiments estimated spine diameter fluctuated on average towards the mean, small spines becoming, on average, slightly larger and large spines slightly smaller (Figure 1(c)). There was no significant difference in the fluctuation over the time course of the experiment (2-way ANOVA size versus time, both apical CA1 pyramidal cells and DG granule cells: significant effect of head size *p* < 0.0001, no effect of time, *p* ~ 0.6, and no interaction, *p* ~ 0.5; *n* = 176 spines in 7 preparations for CA1 cells and 83 spines in 4 preparations for granule cells). For the purpose of illustration, the mean change in spine head size of each group was averaged across all time points (outer limit of shaded region, Figures 1(c), 2(b), and 3(b)) although the relevant time point was used for statistical comparison with test data.

*Effects of Osmolarity*. The eventual aim of the study was to understand the role of different spine types when simultaneously stimulated by an induction protocol that would cause LTP, in this case 25 mM TEA. Addition of 25 mM TEA altered the osmolarity of the solution from 315 mOsm to 365 mOsm. As it is not possible to prevent this increase in osmolarity without changing Na^+^ concentration which would alter the excitability of the system and compromise the LTP, parallel experiments were conducted to assess the contribution of osmolarity to both the slope of the field potentials and the change in spine size. To this end, sorbitol (50 mM) was substituted for TEA, increasing osmolarity (by 50 mOsm without causing LTP) in otherwise identical experiments to the TEA experiments below. Sorbitol caused a transient decrease in field potential slope reaching a minimum level of ~50% of baseline values at around 10 minutes in both CA1 and dentate gyrus which then returned to baseline level by 20 minutes in both areas (Figures 2(c) and 3(c)). The sorbitol was tested in acute slices in both CA1 (*n* = 5) and dentate gyrus (*n* = 13) and the time course confirmed in the dentate gyrus in organotypic slices (*n* = 2). This decrease in synaptic strength was not accompanied by a change in paired-pulse ratio (PPR) and hence was probably not due to a presynaptic effect such as osmotically induced depletion of the readily releasable vesicle pool. We hypothesized that the osmolarity-induced depression may have been associated with a decrease in the size of spines, particularly large spines due to loss of H_2_O down the osmotic gradient. However, in the CA1 region, the decrease in field potential slope was not associated with any decrease in spine size (*n* = 122 spines in 4 preparations). In fact, both small and large spines transiently showed a significant increase in size when compared to controls, peaking at 10 minutes during washout of sorbitol. Large spines consistently returned to baseline by 20 minutes while small spines returned to control levels at a variable rate but always by 60 minutes (Figure 1(d) and dotted lines in Figures 2(b) and 3(b)). In contrast, in the dentate gyrus, increased osmolarity resulted in delayed shrinkage of the large spines peaking with a significant change from control data at 30 minutes. Osmolarity had no significant effect on small spines compared to controls at any time point in dentate gyrus (*n* = 75 spines in 4 preparations).

Hence, the transiently decreased synaptic response resulting from increased osmolarity was accompanied by a trend towards growth of spines rather than shrinkage, particularly in the CA1 region, opposing the expected effect of osmolarity and breaking the usual association between synaptic strength and spine morphology.

### 3.2. Effects of TEA

**Figure 4:**
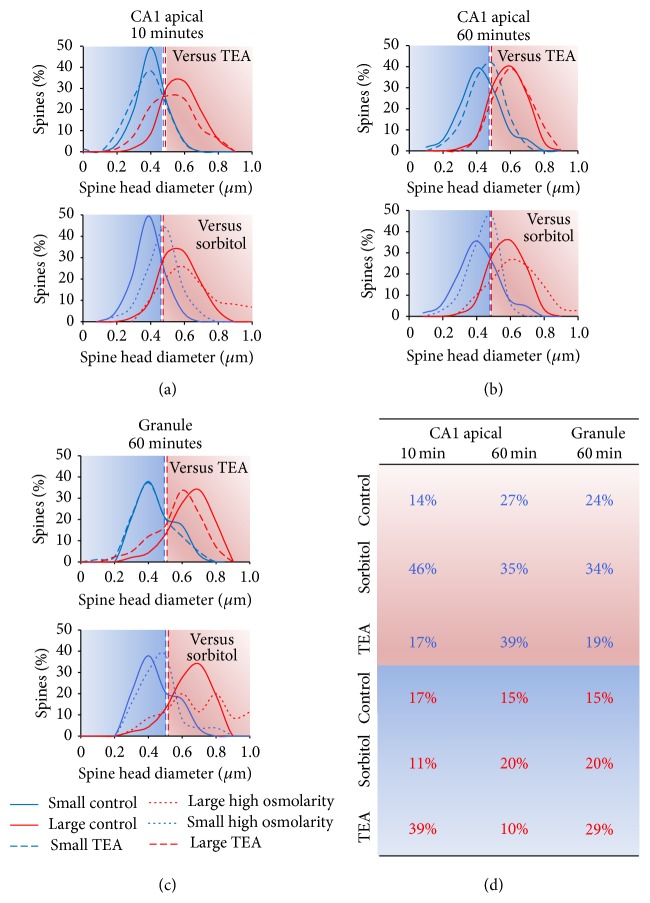
Comparison of small (blue) and large (red) spine head distributions in control versus TEA and control versus sorbitol experiments. ((a), (b)) Distribution of diameters estimated from computed volume measurement of spines in CA1 pyramidal cell apical dendrites (a) 10 and (b) 60 min after application of control (unbroken lines) versus upper panel, TEA (dashed lines), or lower panel, sorbitol (dotted lines), at the same time points. By 60 min, large spines (red) have returned to their original size being no different from control spines, whereas the small spines have grown, indicated by the shift of the distribution (blue) to the right. (c) Dentate granule cell distribution of spine diameters at 60 min. Upper panel: the distribution of small spines (blue) is not greatly affected by TEA (dashed lines) whereas the distribution of large spines (red) shifts to the left showing the persistent decrease in spine head diameter compared to controls (unbroken lines). Lower panel: sorbitol (dotted lines) shows no significant change compared to control (unbroken lines). The blue and red backgrounds represent the diameters defined as small or large, respectively, in the initial category definition at −10 min according to mean diameters (vertical dashed lines). (d) Percentages of spines belonging to each size category (as defined at −10 min) that cross the mean into another category at specified time points after application of TEA or sorbitol. Starting category indicated by font colour (blue text, small; red text, large); final category indicated by background colour (blue, small; red, large). Hence, blue writing on a red background indicates a spine that was initially in the small category but that moved across the threshold to the large category by the time point indicated.

In order to assess the interaction of long-term plasticity and spine size, TEA was applied as a global stimulus to hippocampal slices. TEA blocks potassium channels causing widespread depolarization and glutamate release. The effect of TEA on spines of CA1 pyramidal cells, in which application of TEA consistently causes LTP, was compared to effects in DG granule cells where TEA fails to induce LTP [10]. Effects of TEA differed between the CA1 region and the dentate gyrus in both the electrical and morphological changes observed but in both cases changes in small spines and changes in large spines differed in direction and time course (Figures 2–4). Interestingly, in the presence of TEA, simultaneous changes in the size of large and small spines were never observed. Changes in spine size in the TEA experiments were analysed relative to both control and osmolarity experiments.

#### 3.2.1. CA1 Pyramidal Cells

Changes in spine size were measured in apical dendrites of CA1 pyramidal cells (Figures 2(a) and 2(b), *n* = 176 spines in 7 preparations). Application of TEA immediately caused shrinkage of large spines, reaching a minimum at 10 minutes (*p* < 0.01) but returning to baseline by 30 minutes. Note that, as outlined above, this decrease in the size of large spines could not be explained by a response to a change in osmolarity because the small change observed when in sorbitol was in the opposite direction to the change caused by TEA (*p* < 0.0001 sorbitol versus TEA, 10 min).

In contrast to the shrinkage of large spines, within the same dendritic segments, small spines showed not only a different direction of change but an entirely different time course. Initially, in the presence of TEA and over the following 20 minutes of washout, TEA caused a similar fluctuation in small spines to control conditions. Hence, addition of TEA opposed the growth of small spines caused by increased osmolarity (*p* < 0.0001, 10 min; *p* < 0.05, 20 min). However, when the effect of osmolarity washed out and the large spines had returned to control levels, small spines began to grow significantly and, by 60 minutes, showed a 4-fold greater increase on average than that shown by small spines either in the control condition (*p* < 0.0001) or in the presence of sorbitol (*p* < 0.05). Effect on the distribution of spine sizes of sorbitol or TEA versus control conditions at 10 and 60 minutes is illustrated in Figure 4.

In order to investigate how changes in spine morphology were related to TEA-induced changes in synaptic strength, fEPSPs were recorded under conditions as close as possible to those of the imaging experiments (Figure 2(c); see Section 2). The addition of TEA to the bath initially resulted in a brief increase in fEPSP_slope_ which was apparently largely presynaptic in origin, as it was paralleled by a drop in PPR, usually indicative of an increase in release probability. This was followed by substantial depression of the measured postsynaptic response with fEPSP_slope_ decreasing over 10 min. This depression can largely be explained by the effect of increasing osmolarity as it is mirrored by the application of sorbitol. A tendency of the presynaptic volley to widen and decrease suggests a loss of excitability of the presynaptic axons; however, considering the apparent increase in release probability, this was unlikely to be the major reason for the depression (Figure 2(c), inset). The decrease in PPR lasted for 30 minutes after TEA application and could be largely attributed to a presynaptic effect of TEA, rather than osmolarity, as this was not seen in sorbitol control experiments. As the effect of osmolarity declined, the size of the postsynaptic response returned towards baseline eventually revealing potentiation of the fEPSP with an ongoing contribution from increased presynaptic release. The PPR returned to baseline level by about 30 minutes at which time the synaptic response settled to a plateau of potentiation (125 ± 9%, *n* = 7; *p* < 0.05; paired *t*-test lasts 10 minutes versus baseline) presumably mediated postsynaptically.

It is notable that while the early loss of electrical response could be wholly attributed to the effect of increased osmolarity, the decrease in the size of large spines was entirely TEA dependent as was the change in PPR. Moreover, these two purely TEA-induced effects occur with a similar time course suggesting that the transient shrinkage of large spines could be a short-term homeostatic response to the increased release probability and general spill-over of glutamate resulting from the TEA-induced global stimulation. This would have the protective effect of preventing an excessive postsynaptic response. Note that the decrease in PPR suggests an increase in release probability that would be expected to cause an increase in the postsynaptic response if it were not for opposing postsynaptic factors. Moreover, the final stable potentiation by 30 min after TEA application, once PPR had returned to baseline, was also consistent with consolidation of a postsynaptic change by the delayed growth of small spines.

#### 3.2.2. Dentate Granule Cells

When similar imaging experiments were carried out in DG granule cells in organotypic slices, the pattern of change was different from spines in the apical dendrites of CA1 pyramidal cells (Figure 3, *n* = 167 spines in 10 preparations). TEA had no effect on small spines which behaved similarly across the time course of the experiment, tending to be even more stable than when osmolarity was changed in the absence of TEA (*p* < 0.05, 10 min). In contrast, large spines showed an immediate small but significant decrease in size on application of TEA but, unlike in the CA1 region, the decrease persisted throughout the experiment being statistically significant compared to control at all time points (*p* < 0.05 at 0, peaking at *p* < 0.0001 at 20 min). This result was however difficult to interpret as, with the exception of the 10-minute time point (*p* < 0.01), the effect of TEA on large spines was similar to the effect of sorbitol-induced increased osmolarity.

TEA however clearly affected the small and large spines differently (interaction between treatment and size, *p* = 0.01).

The spine response was again reflected in the field recordings. As suggested by the spine morphology, application of TEA induced LTD in the DG granule cells of organotypic slices (Figure 3(c), *n* = 7). Thus, even after washout and recovery from the extreme depression caused by the presence of TEA, the stable plateau reached by 20–30 min was significantly lower than baseline (73 ± 3.4%, *n* = 9; *p* < 0.0005). To assess whether the difference in effect of TEA on field response in the dentate gyrus versus the CA1 region was due to the organotypic preparation versus the acute slice, we also compared the effect of TEA in acute slices in dentate gyrus and tetanus-induced LTP in both CA1 and dentate gyrus. In both cases, robust LTP was measured in the CA1 region and no LTP was observed in the dentate gyrus, although responses in acute slices returned to baseline, rather than showing the long-term depression seen in organotypic slices (data not shown). The effects of osmolarity were however similar in both preparations. Hence, in dentate gyrus where TEA failed to cause LTP, the delayed response of small spines was absent whereas large spines still showed a similar homeostatic response to the strong stimulus, albeit not recovering once the stimulus was removed.

## 4. Discussion

In the present study, application of TEA is used as a tool to stimulate many spines simultaneously in order to investigate how they interact when both protective homeostatic and long-term potentiating responses would be expected; specifically, we aim to tease out whether different types of spines subserve different functions. To this end, we studied spines both in the CA1 region where TEA causes LTP and in the dentate gyrus where LTP was absent under these conditions.

In earlier studies, spines have frequently been defined into categories such as filopodia, stubby, thin, and mushroom according to head size, head size as a ratio to neck diameter, and length of spine or other criteria [18]. In particular, transitions between thin and mushroom spines have been suggested to play a role in synaptic plasticity (for review, see [19]). These categories have been very useful particularly in relation to electron microscopy studies that allow resolution of the presence of specialized endoplasmic reticulum and other features of the spine (for review, see [20]). However, whether there is a clear distinction or a continuum between spine types is not clear and certainly with confocal microscopy, accurate measurement of spine necks is not possible and the size of spine heads shows a near-Gaussian distribution. Hence, many spines would fall into an area between thin and mushroom relying on subjective judgments for definition. In the present study, we have restricted analysis to spines with clear heads hence excluding stubby spines or filopodia. The division between small and large spines would be roughly equivalent to thin and mushroom spines, respectively, but the clear cut-off at mean diameter allows an entirely objective division that has recently been preferred in light microcopy studies [7]. The analysis here includes all spines that could be well fitted by Imaris. This excludes spines too small for resolution and some very large spines (see Section 2). It is possible that additional changes of interest would be seen if such spines could be included.

It has been previously reported that when stimulation is applied to single spines in the CA1 apical dendrites, using repetitive photolysis of MNI-glutamate, response to glutamate and spine head size increases immediately, independent of the starting size [7]. However, while this growth and the resulting synaptic potentiation can be long-lasting for small spines, it is only transient in large spines. This shows that stimulation of individual synapses affects small and large spines differently but does not clarify the question of different functional entities, as it may reflect a continuum limited by the maximum head size that an individual spine can maintain. Moreover, single spines would rarely be activated in isolation under physiological or indeed pathological conditions and the response to stimulation of a spine may be influenced by the responses of neighboring spines.

Here we report that responses in large and small spines can be functionally differentiated when stimulated simultaneously. Our results in apical dendrites of CA1 pyramidal cells are in agreement with a previous TEA study in CA1 of hippocampal organotypic slices, which also showed that long-term changes are mostly related to small spines [9] as is also true for individual spine stimulation [7]. However, Hosokawa and colleagues [9] only investigated effects 2 hours after TEA application and so the shrinkage of large spines and stability of small spines observed here, in the presence and during washout of TEA, would have been missed. We suggest that the most likely reason for the immediate shrinkage, which reverses during TEA washout, is a homeostatic response to overstimulation. Although increased osmolarity has been used to cause glutamate release in single boutons in culture [21, 22], these studies used 6–10-fold the osmolarity used in the present study and demonstrated that neighbouring spines, which would have been exposed to lower level osmolarity changes, were not affected. The suggestion that changing osmolarity by 50 mOsm is unlikely to have changed glutamate release is supported by the stability of the PPR when sorbitol was added in the osmolarity controls for the present study. In another study, using electron microscopy, Stewart and coworkers were unable to detect changes in spine volume one hour after TEA washout [23]. In this study, the stimulus was more extreme (25 mM TEA applied for 20 min in the presence of 10 mM Ca^2+^ and 5 mM K^+^ and in the absence of Mg^2+^) and again, only a single time point was observed. Clearly under such conditions recovery from stimulation would be likely to occur later and so, even if similar effects occurred to those observed in the present study, it is possible that the point of sampling happened to coincide with the time at which large spines recovered and small spines had not yet started to grow.

The delay observed here, before small spines grow, is also different from the immediate growth reported when a small spine is stimulated individually [7]. This suggests that the delay is the result of interaction between spines when they are simultaneously stimulated. The delay is particularly remarkable in the light of increased osmolarity apparently causing a transient increase in the size of small spines in the absence of changes in large spines, suggesting that either the TEA-induced change in large spines or other effects of TEA actively prevent this change. Possibly the head size of small spines would also be seen to decrease similarly to the effect seen in large spines if the osmolarity could be kept constant.

Considering the depressive effect of osmolarity on synaptic transmission in both CA1 and dentate gyrus, it is interesting that this is not reflected in spine size. This is an example where the generally close link between spine size and synaptic response becomes dissociated. One possibility is that the change in osmolarity alters the geometry of the synapse such that pre- and postsynaptic sides temporarily lose their close apposition. This would mean that, even with increased release suggested by the changed PPR in the presence of TEA, the response would remain depressed.

The application of TEA causes many effects including broadening of the action potential, which likely influences our electrophysiological measurement of synaptic response, so that the relative contributions of pre- and postsynaptic factors to the depression of the electrophysiological responses in the presence of TEA are hard to assess. However, the TEA-dependent decrease in PPR in both regions over the first 20 minutes after TEA application would be expected to correlate with an increase in the response. Hence, the depression associated with the decrease in the size of large spines is likely to be underestimated. The field recordings do serve to indicate a time course of the maximal acute effects of TEA however and the substantial shrinkage of the large spines strongly suggests a postsynaptic component in organotypic slices under these conditions. It is perhaps surprising that the increased osmolarity does not contribute to this shrinkage, although it clearly is a factor in the transient synaptic depression. Indeed, shrinkage would be the expected effect of osmolarity as H_2_O moves towards the hyperosmotic compartment; however, dendritic spines appear to be able to resist any osmolarity-induced shrinkage within these limits.

The electrophysiological responses confirm the difference in the effect of TEA on CA1 pyramidal cells and DG granule cells. It is interesting to note that DG granule cells show a similar immediate response to TEA but the depression is greater and shows a very different long-term response both electrically and in the changes seen in spine morphology. The question arises whether the initial extreme depression is irreversible in some large spines in the dentate gyrus possibly reflecting lack of recovery of function in a subset of synapses rather than a true long-term depression across the population. The present observations that conditions causing LTP in the CA1 region do not cause potentiation in the dentate gyrus are in agreement with previous electrophysiological studies in acute rat hippocampal slices [10]. Note that if stimulated separately, the synapses of the medial and lateral perforant path have different characteristics in relation to short-term plasticity but behave similarly in terms of tetanus-induced LTP when recorded in the absence of GABA_A_ receptor antagonists [24]. Moreover, in organotypic slices, these pathways are likely to be less clearly defined and use of chemical LTP will stimulate all pathways equally. Hence, while both pathways were stimulated, the results were pooled in this study.

## 5. Conclusions

In conclusion, we suggest that, in the CA1 region, a subset of spines has specific functions that do not represent a continuum across the spectrum of spine morphologies. In both CA1 and DG, we propose that it is large spines that are important for immediate short-term homeostatic protection while, at least in the CA1 region, the delayed growth of small spines follows the increase in synaptic response, stabilizing the alteration in AMPA receptors that may underlie learning and memory. Moreover, throughout this study in both CA1 and DG, small and large spines never change simultaneously. Occurrence of LTD and LTP depend strongly on the Ca^2+^ dynamics in individual spines and have previously been reported to be mutually inhibitory via the phosphorylation and dephosphorylation of glycogen synthase kinase-3 [25, 26]. Such a mechanism may be involved in the interactions reported here. Moreover, under normal physiological stimuli onto individual spines, Ca^2+^ transients are large and rapid but restricted to the spine [27], whereas, under a strong stimulus such as that used here, the diffusion of Ca^2+^ between large and small spines may contribute to communication between spines of different sizes [28].

The network-wide stimulation used in this study could be compared to the pathological effects of ischemia or epilepsy rather than the more subtle stimuli required for the specific laying down of memory. These observations could thus be important in the well-established interactions that occur between such pathological processes and memory [29]. Moreover, the separation of these effects could have important implications in relation to the link between acute pathological insults and eventual long-term effects in Alzheimer's disease or other neurodegenerative conditions. We suggest that the strong synaptic depression mediated by shrinkage of large spines during and immediately after the application of TEA and the resulting delay in small spine growth combine to protect the neurone by decreasing the influx of Ca^2+^ and the damage that this could cause, short- and long-term, if uncontrolled (see [30] for review of implications in neurodegeneration). Indeed some of the earliest changes suggested to occur in Alzheimer's disease are related to Ca^2+^ homeostasis [31]. Large spines are known to be anatomically different from small spines, containing considerably more smooth endoplasmic reticulum, often associated with a spine apparatus that is not present in small spines [32]. Although the reason for these differences is not clear, such specialized organelles could be essential if large spines serve specific functions in relation to protecting the neurone from excessive Ca^2+^ influx in pathological situations. It may therefore be possible to target the failure of such specialized spines selectively without changing the memory supporting functions of the small spines.
